# Post-Graduate Study

**Published:** 1886-06

**Authors:** 


POST-GRADUATE STUDY.

Thoughtful men in dentistry are beginning to consider the possibility of a higher professional education than that offered by either the average medical or dental school. They realize the fact that if we are to diligently cultivate that particular portion of the field of science which we claim as our rightful heritage, if we are to take rank as scientific men and to obtain recognition from the purely scientific world, we need more perfect methods and to rise to a yet higher plane. The general information obtained in our schools is not sufficient. We must make advancement in abstract science as pertaining to our specialty, and not spend our whole time in applied study. Our schools are, from year to year, raising their standard of graduation, and perhaps advance as fast as they are warranted in doing by the average state of professional opinion. But they do not go far enough for the real students, who are demanding something more, and who crave a yet deeper erudition than that which is indexed by the D. D. S. or the M. D.

There are many men whose early opportunities were restricted by their environments, who entered the profession of dentistry by the back door, and who are to-day without the distinctive degree which indicates scholastic training. Some of these men will be content with cheap diplomas, issued by too accommodating cheap schools, and obtained by only nominal labor. There are others whose aim is not the possession of a diploma, but the acquisition of real knowledge. They realize the fact that a diploma is not a perfect index of the amount of information obtained, and sigh for something further. Others, having graduated regularly, desire to continue a systematic course of study, and to keep up with the advance of scientific knowledge. There are many allied branches of medical or dental learning which they wish to study.

What shall be done for these men? They are engaged in practice, have families dependent upon them, have many ties which bind them to home, and hence it is impossible for them to attend any of the higher schools, or to go abroad for study. Is it not practicable to establish a grade of study to be pursued at home, to mark out a systematic course to be pursued under competent instructors, the tuition to be obtained through correspondence or by the publication of courses of lectures on definite themes, somewhat after the manner of the Chautauqua Literary Course, but modified to suit the exigencies demanded?

At the late meeting of the Illinois State Dental Society, a very thoughtful paper bearing upon this subject was presented by Dr. J. D. Moody, of Mendota, and it elicited the hearty approval of the intelligent body of men before whom it was read. This journal will give an abstract of that paper in due time, and hopes for something further upon the subject from its competent author. It is a theme worthy the earnest consideration of any man, and we commend it to the thoughtful reader, and shall be glad to receive communications concerning it. Be assured it is a want that must soon be provided for. Dentistry is a young and pushing science, and it will not long be content with the present grade of scholastic requirements. It is to be hoped that the subject will be presented before the coming meeting of the American Dental Association, that an expression of the views of some of our best men may be obtained.

